# Supplementary material for: Deletion of wfs1 Impairs Oligodendrocyte Precursor Cells Dorsal Distribution and Myelination Through the wfs1-hmgcs1 Axis in Zebrafish
Source: Biology (Basel). 2026 Aug 16;15(16):1402. doi: 10.3390/biology15161402 (PMC13510151; doi:10.3390/biology15161402)
Supplement: Supplementary file 1 [file biology-15-01402-s001.zip › File S1-Transcriptome sequencing data and differentially expressed genes (DEGs) in wfs1-- zebrafish at 5 dpf/2.KEGG/wfs1KOvsWT/ALL/wfs1KOvsWT.all_KEGGdot.pdf]

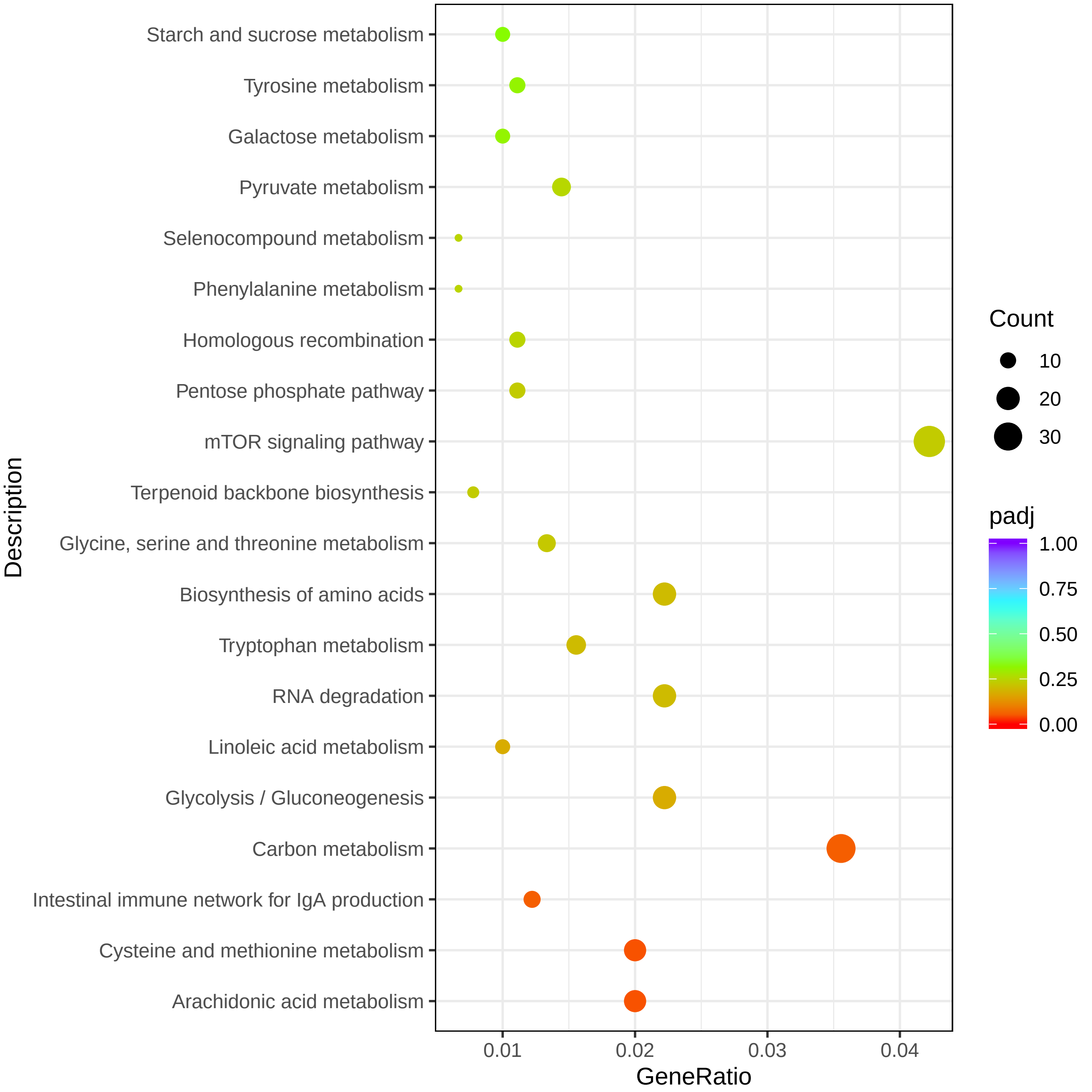

Starch and sucrose metabolism
Tyrosine metabolism
Galactose metabolism
Pyruvate metabolism
Selenocompound metabolism
Phenylalanine metabolism
Homologous recombination
Pentose phosphate pathway
mTOR signaling pathway
Terpenoid backbone biosynthesis
Glycine, serine and threonine metabolism
Biosynthesis of amino acids
Tryptophan metabolism
RNA degradation
Linoleic acid metabolism
Glycolysis / Gluconeogenesis
Carbon metabolism
Intestinal immune network for IgA production
Cysteine and methionine metabolism
Arachidonic acid metabolism
Description
0.01
0.02
0.03
0.04
GeneRatio
Count
10
20
30
padj
1.00
0.75
0.50
0.25
0.00
